# The GDF11 Promotes Nerve Regeneration After Sciatic Nerve Injury in Adult Rats by Promoting Axon Growth and Inhibiting Neuronal Apoptosis

**DOI:** 10.3389/fbioe.2021.803052

**Published:** 2022-01-04

**Authors:** Junhao Lin, Jie Shi, Xiang Min, Si Chen, Yunpeng Zhao, Yuanqiang Zhang, Lei Cheng

**Affiliations:** ^1^ Department of Orthopaedic, Qilu Hospital, Cheeloo College of Medicine, Shandong University, Jinan, China; ^2^ Cheeloo College of Medicine, Shandong University, Jinan, China; ^3^ NHC Key Laboratory of Otorhinolaryngology, Qilu Hospital, Cheeloo College of Medicine, Shandong University, Jinan, China; ^4^ Department of Health Management Center, Qilu Hospital, Cheeloo College of Medicine, Shandong University, Jinan, China; ^5^ Department of Neurosurgery, Qilu Hospital, Shandong University, Jinan, China

**Keywords:** sciatic nerve injury, nerve regeneration, lentiviral vector, growth differentiation factor 11, smad pathway

## Abstract

**Introduction:** Sciatic nerve injury is a common injury of the nervous system. Stem cell-based therapies, drug-based therapies and rehabilitation physiotherapy therapies are currently available, but their limited therapeutic efficacy limits their use. Here, we aimed to explore a novel lentiviral-based gene therapeutic strategy and to elaborate its mechanism.

**Materials and Methods:** Recombinant GDF11 protein was used for the *in vitro* treatment of dorsal root ganglion (DRG) cells. Lentivirus was used to construct a vector system for the *in vivo* expression of GDF11. The nerve conduction function was detected using action-evoked potentials at different time periods, and the regulatory effect of nerves on target organs was detected by weighing the gastrocnemius muscle. Immunofluorescence of NF200 and S100 was used to show the regeneration of the sciatic nerve, and myelin and Nissl staining were performed to observe the pathological features of the tissue. Western was used to validate signaling pathways. The expression of related genes was observed by qPCR and Western blotting, and cell apoptosis was detected by flow cytometry.

**Result:** GDF11 promotes the axonal growth of DRG cells and inhibits DGR cell apoptosis *in vitro*. GDF11 acts by activating the Smad pathway. GDF11 promotes the recovery of damaged sciatic nerve function in rats, the regeneration of damaged sciatic nerves in rats, and myelin regeneration of damaged sciatic nerves in rats. GDF11 also exerts a protective effect on neuronal cells in rats.

**Conclusion:** Based on the present study, we conclude that GDF11 promotes axonal growth and inhibits DRG cell apoptosis *in vitro* through the Smad pathway, and lentivirus-mediated GDF11 overexpression *in vivo* can promote the recovery of sciatic nerves after transection by promoting axonal growth and inhibiting neuronal apoptosis in the spinal cord.

## Introduction

Peripheral nerve injury is more common than central nervous system injury but has not received sufficient attention. Peripheral nerve injury often leads to heavy psychological stress and a decline in quality of life and thus results in a heavy social and economic burden (Geuna, 2015; Liu et al., 2018). Peripheral nerve transection is one of the most common peripheral nerve injuries, but its current treatment does not yield satisfactory effects. Transection injuries are caused by a cutting object (for example, knife wounds, broken glass, metal shards, chainsaw blades, wood splinters, and animal bites) (Wang C.-X. et al., 2018). Although neurons have a limited ability to regenerate, axons have a great potential for regeneration (Guo et al., 2016; He et al., 2016; Fang et al., 2019; Yang et al., 2020), and peripheral nerve damage is thus more likely to heal than central nervous system damage (Gu et al., 2014; Geissler and Stevanovic, 2019). The sciatic nerve is accompanied by nerve degeneration and nerve regeneration after injury. Although the specific molecular mechanism of sciatic nerve degeneration and regeneration is not very clear, it is generally believed that this is a complex series of events, which includes local macrophage activation after injury, the attraction of circulating immune cells participating in necrosis (Shi et al., 2018; Fan et al., 2019; Kalinski et al., 2020) and neural axon degeneration and atrophy (Yu et al., 2018) due to an abnormal neuronal cell body leads to increased Schwann cell apoptosis increased (Jia et al., 2020; Yardim et al., 2021), the demyelination of nerve fibers and the local proliferation of fibroblasts to induce scar formation (Que et al., 2013). During this process, the body will gradually adjust to these adverse factors such that the injured nerve slowly undergoes incomplete repair.

Neurotrophic factors are cytokines that regulate the growth and development of the nervous system. Endogenous neurotrophic factors can inhibit neuronal apoptosis (Xue et al., 2021), promote neuronal axon growth and regulate neuronal differentiation (de León et al., 2021). Different neurotrophic factors play different roles, and these roles can be synergistic or even opposite. At present, the treatment of peripheral nerve injury mainly includes stem cell therapy (Zhao et al., 2019; Xia et al., 2020; Zhou et al., 2020; Guo et al., 2021a; Guo et al., 2021b; Yuan et al., 2021), biomaterial research (Shang et al., 2018; Li G. et al., 2019; Tang et al., 2019; Guo J. et al., 2020; Han et al., 2020; Lin et al., 2020), and injection of bioactive drugs (Li et al., 2018; Li D. et al., 2019; Pukos et al., 2019; Cai et al., 2021; Yang et al., 2021), among others. Many studies have shown that the injection of neurotrophic factors can promote the recovery of nerve function after peripheral nerve transection, promote the growth of nerve axons, inhibit neuronal apoptosis and promote nerve regeneration (Gris et al., 2004; Gong et al., 2017; Waqas et al., 2017). However, these methods themselves may cause secondary iatrogenic injuries, which limits their application in the treatment of peripheral nerve injuries. Therefore, the identification of an effective cytokine for promoting nerve regeneration is very important.

Growth differentiation factor 11 (GDF11) is a transforming growth factor β (TGFβ) protein. Some studies have suggested that GDF11 plays a negative role in the regulation of the normally developing nervous system (Kim et al., 2005; Kong et al., 2020), but other studies have shown that GDF11 can inhibit cell apoptosis (Mei et al., 2016), promote axon and synaptic development (Hocking et al., 2008; Augustin et al., 2017), induce neurovascular regeneration and improve nervous system function in elderly individuals (Katsimpardi et al., 2014). However, due to the half-life of the injected cytokines and neurotrophic factors, their effects in the body are relatively short, multiple injections are needed, or their high cost limits their development.

To find a more effective treatment strategy for sciatic nerve injury, our study confirmed the protective effect of GDF11 on neurons *in vitro*, and in a further study, we used a lentiviral vector to induce GDF11 overexpression and thus create and maintain a local microenvironment conducive to nerve regeneration. Lentivirus is a safe *in vivo* expression vector that is currently widely used (Anger-Gora et al., 2021; Reyne et al., 2021). Specifically, we found that lentivirus can transfer the GDF11 gene into the peripheral nervous system and overexpress GDF11 to continuously produce a large amount of GDF11 locally and thus promote the recovery of nerve function after peripheral nerve transection. The *in vivo* transfection of lentiviral GDF11 can effectively avoid secondary iatrogenic injury, has the advantage of reducing the treatment costs, and provides a new treatment strategy for peripheral nerve injury. Based on these findings, we hope to find a strategy for regulating the microenvironment at the site of injury, promote the repair of an injured sciatic nerve, and explore its mechanism to provide a new vision for exploring the mechanism of sciatic nerve injury and developing treatment strategies.

## Materials and Methods

### Animals

Wistar rats were obtained from the Laboratory Animal Centre of Shandong University. A total of 40 male Wistar rats were randomly divided into two groups (each with 20 rats): a control group (rats were injected with lentiviral virus after sciatic nerve transection) and a GDF11 group (rats were injected with lentiviral GDF11 virus after sciatic nerve transection). All animals were maintained under controlled light/dark (12/12 h), temperature (22°C), and humidity (60%) conditions. Food and water were available ad libitum. The pain and numbers of the experimental rats were reduced as much as possible while still meeting the experimental objectives.

### Ethics Statement

The protocol was approved and monitored by the International Guiding Principles for Animal Research, as stipulated by the World Health Organization and as adopted by the Laboratory Animal Centre of Shandong University.

### Surgical Procedures

**FIGURE 1:**
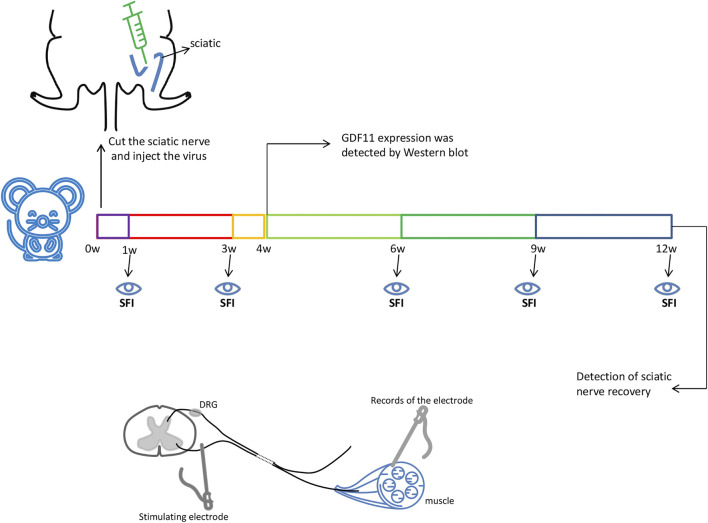
The sciatic nerve of rats was surgically cut off 8mm, and then injected with GDF11 lentivirus or control virus immediately. The sciatic nerve of rats was taken 4 weeks after surgery to detect the expression of GDF11, and SFI values were measured at 1, 3, 6, 9, and 12 weeks, respectively. The sciatic nerve recovery was measured at 12 weeks. Stimulating electrodes were used at the proximal end of the injury, and evoked potentials were detected at the distal muscle.

To simulate sciatic nerve injury in rats, a rat sciatic nerve transection model was used in this study. The operation was performed under deep anesthesia through the intravenous injection of pentobarbital sodium (20 mg/ kg). The surgical site was first shaved and prepared with 75% surgical alcohol. The operation was performed under aseptic conditions as much as possible, and the left sciatic nerve was then exposed through a gluteus cleavage incision and severed with microsurgical scissors. All animals were immediately subjected to a tension-free suture of the transected sciatic nerve with a nerve pontine (silicone tube, Shandong Institute of Medical Instruments, Shandong, China) to obtain an 8-mm gap between the proximal and distal stump. Subsequently, 20 µL of control lentiviral or lentiviral GDF11 virus (5.5 × 10^8^ TU/ ml) was injected into the injured area, the muscle tissue was replaced, the skin was sutured, and the animals were returned to their cages (Figure 1), Nerves within 1 cm of injury were used to detect subsequent experimental indicators.

### Culture of Dorsal Root Ganglion (DRG) Cells

Two-to three-day-old newborn Wistar rats (provided by the Animal Experimental Center of Shandong University) were used for extraction of the DRG, and the rats were removed and killed. The rats were then impregnated in 75% ethanol and disinfected for 5 min. The DRG was carefully removed, and the residual nerve roots were resected with a stereoscopic microscope. DRG cells were then plated on PDL-coated slides and placed in 6- or 24-well plate with neurobasal medium containing 2% B27, 0.3% L-glutamine and 1% penicillin-streptomycin in an incubator at 37°C with 5% CO_2_ and 95% humidity. The medium was changed every 3 days, and recombinant GDF11 (10 ng/ ml) was added on the first day of culture.

### Western Blot Analysis

Cell or tissue lysates were prepared in RIPA buffer (Beyotime Biotechnology, Shanghai, China). After the total protein concentrations were detected using a bicinchoninic acid (BCA) protein assay (Beyotime, China), an equal amount of protein from each sample was resolved by SDS-PAGE on 10% SDS-polyacrylamide gels and transferred to a polyvinylidene difluoride (PVDF) membrane for immunoblot analyses. The membranes were incubated with the following primary antibodies overnight at 4°C: rabbit anti-GDF11 (1:1000; Abcam, ab124721, United States), rabbit anti-Bax (1:1000; affinity Biosciences, AF0120, United States), rabbit anti-caspase3 (1:1000; affinity Biosciences, DF6879, United States), rabbit anti-Bcl2 (1:500, Proteintech, 12789-1-AP, United States), rabbit anti-smad2/3 (1:1000, CST, 8685S,United States), rabbit anti-p-smad2/3 (1:1000, CST, 8828S, United States), rabbit anti-smad1/5/8 (1:1000, abcam, ab66737, United States), rabbit anti-p-smad1/5/8 (1:1000, CST, 13820S, United States), and mouse anti-β-actin (1:10000; CST, 8H10D10, United States). The unbound antibodies on the PVDF membranes were washed, the secondary antibody (1:10000, CST, 5470P, United States), and (1:10000, CST, 5366P, United States) was added, and the bands were visualized with an infrared laser imaging system (Li-COR, United States). ImageJ software (National Institutes of Health, United States) was used for densitometry analysis.

### RT-PCR

Total RNA was extracted from the tissue or cells using the TRIzol (Invitrogen) reagent according to the manufacturer’s instructions. The RNA samples were reverse-transcribed after the genomic DNAs were removed using a reverse transcription kit (Takara, Tokyo, Japan). The cDNA product was stored at –20°C. For real-time PCR, a 10-μL reaction mixture containing 50 ng of cDNA was used. The reactions were performed with a PCR instrument (ABI-7900, United States). The primer sequences were as follows: β-actin forward, 5′-CTC​TGT​GTG​GAT​TGG​TGG​CT-3′, and reverse: 5′-CGC​AGC​TCA​GTA​ACA​GTC​CG -3′; Bcl2 forward, 5′-CTG​AGT​ACC​TGA​ACC​GGC​AT-3′, and reverse, 5′-ATA​TAG​TTC​CAC​AAA​GGC​ATC​CCA​G-3′; Bax forward, 5′-TTT​GCT​ACA​GGG​TTT​CAT​CCA​GG-3′, and reverse: 5′-CGC​TCA​GCT​TCT​TGG​TGG​AT-3′; and caspase3 forward, 5′-CGG​ACC​TGT​GGA​CCT​GAA​AA-3′, and reverse, 5′-CGG​CCT​CCA​CTG​GTA​TCT​TC-3′.

### HE Staining

Twelve weeks after injury, sciatic nerve specimens from the injured area were stained with hematoxylin-eosin (HE). The slices were then dehydrated with 70 and 100% gradient ethanol and sealed with polyxylene transparent adhesive for 10 min. The stained sections were examined under a microscope (BX-51, Olympus).

### Nissl Staining

After dewaxing and hydration, Nissl staining was performed with 0.5% cresyl violet solution for 1 min. The stained sections were examined under a microscope (BX-51, Olympus). The number of normal neurons in the spinal cord area was counted by two pathologists.

### IF Analysis

IF staining of sciatic nerve tissue was performed with antibodies against various markers to evaluate the recovery of the sciatic nerve after transection. The tissue was incubated with the anti-Schwann cell marker antibodies S100 (1:100, Abcam, ab52642, United States) and neurofilament 200 (1:400; CST, 2836, United States) for 2 h at 37°C. The samples were then washed 3 times with phosphate-buffered saline and treated with anti-rabbit DyLight-488 (1:200; Abbkine, A23220, United States) and anti-mouse DyLight-594 antibodies (1:200; Abbkine, A23410, United States) for 1 h at 37°C. Subsequently, 40,6-diamidino-2-phenylindole (DAPI) counterstaining was performed for the visualization of cell nuclei. S100 is a Schwann cell marker, NF-200 is a marker for large myelinated A-fiber neurons, and DAPI serves as a marker for the number of nuclei in the captured images. For cellular immunofluorescence, cultured cells were fixed with 4% paraformaldehyde and stained with Neurofilament 200 (1:400; CST, 2836, United States) at 37°C for 2 h. The samples were then washed 3 times with phosphate-buffered saline and treated with anti-mouse Dylihgt-488 (1:200; Abbkine, A23210, United States) for 1 h at 37°C, and 40,6-diamidino-2-phenylindole (DAPI) counterstaining was performed for the visualization of cell nuclei. Images were acquired with a fluorescence microscope (Leica DM5500B) and a digital camera (Leica DFC345 FX, Leica Application Suite X software) and then analyzed using ImageJ software (version 1.45s, National Institute of Health, United States).

### Flow Cytometric Analysis

Cells in a 6-well plate were washed three times with cooled PBS and then collected in a centrifuge tube for further operation. An Annexin V-FITC/PI kit (E-CK-A211, Wuhan, China) was used to determine whether the cells were apoptotic. The cells were resuspended in 1 × binding buffer at a concentration of 1 × 10^6^ cells/mL. Subsequently, 100 µL of this solution was transferred into a 5-ml culture tube, and 5 µL of FITC Annexin Ⅴ (or PE Annexin Ⅴ) and 5 µL of PI were added. The cells were gently vortexed and incubated for 15 min at room temperature in the dark, and 400 µL of 1 × binding buffer was then added to the tube. Flow cytometry was conducted within 1 h with a BD Accuri C6 Plus flow cytometer (San Diego, CA, United States), and the data were analyzed using FlowJo V10 software (BD, San Diego, CA, United States).

### Target Muscle Weight

Twelve weeks after surgery, the animals were euthanized, and the gastrocnemius muscle from the operated limbs of three rats in each group was exposed, dissected, removed and weighed immediately as a simple and direct measure of the effect of GDF11 on the target muscle and nerve function.

### Functional Analysis

For the assessment of sciatic nerve recovery, the degree of recovery was monitored by evaluating the walking patterns of the hind limbs to obtain the sciatic function index (SFI), as described by Bain et al. At weeks 1, 3, 6, 9, and 12 after surgery, three random rats of each group were subjected to a walking track assay, and the SFI was measured based on a previously described protocol. The paw length and toe spreads were measured and used to calculate the SFI using the following formula: SFI = 109.5 (ETSNTS)/NTS38.3 (EPLNPL)/NPL +13.3 (EITNIT)/NIT8.8. In this equation, EPL indicates the operated experimental paw length, NPL is the normal paw length, ETS refers to the operated experimental toe spread, which represents the distance between the first and fifth toes, NTS is the normal toe spread, EIT represents the operated experimental intermediary toe spread, which represents the distance between the second and fourth toes, and NIT is the normal intermediary toe spread. A value of 100 indicates total impairment, and a value of 0 indicates normal or complete recovery.

### Motion-Evoked Potential

Under aseptic conditions, the skin of the injured thigh of the rats was cut open to expose the sciatic nerve, and the sciatic nerve was stimulated with a stimulation electrode at an intensity of 0.65 mA. At the same time, the recording electrode was inserted into the gastrocnemius muscle, and the reference electrode was inserted into the healthy gastrocnemius muscle. An intraoperative neuromonitor (Endeavor CR, United States) was used to record the evoked potential.

### Transmission Electron Microscopy

The nerve tissue was fixed with 2% glutaraldehyde in sodium dimethyl arsenate buffer (0.1 M, pH 7.2) overnight at 4°C and then fixed with 1% osmium tetroxide solution for 1 h. Digital images were acquired with a JEM-1230 high-contrast TEM and soft scan imaging system (JEOL, Tokyo, Japan).

### Statistical Analysis

All the data are presented as the means ± SDs. The statistical significance of the differences was analyzed by *t*-test or one-way ANOVA. *p* < 0.05 was considered to indicate statistical significance. GraphPad Prism eight software (GraphPad Software, La Jolla, CA, United States) was used for all statistical analyses.

## Results

### GDF11 Inhibits the Apoptosis of DGR Cells *in vitro*

**FIGURE 2:**
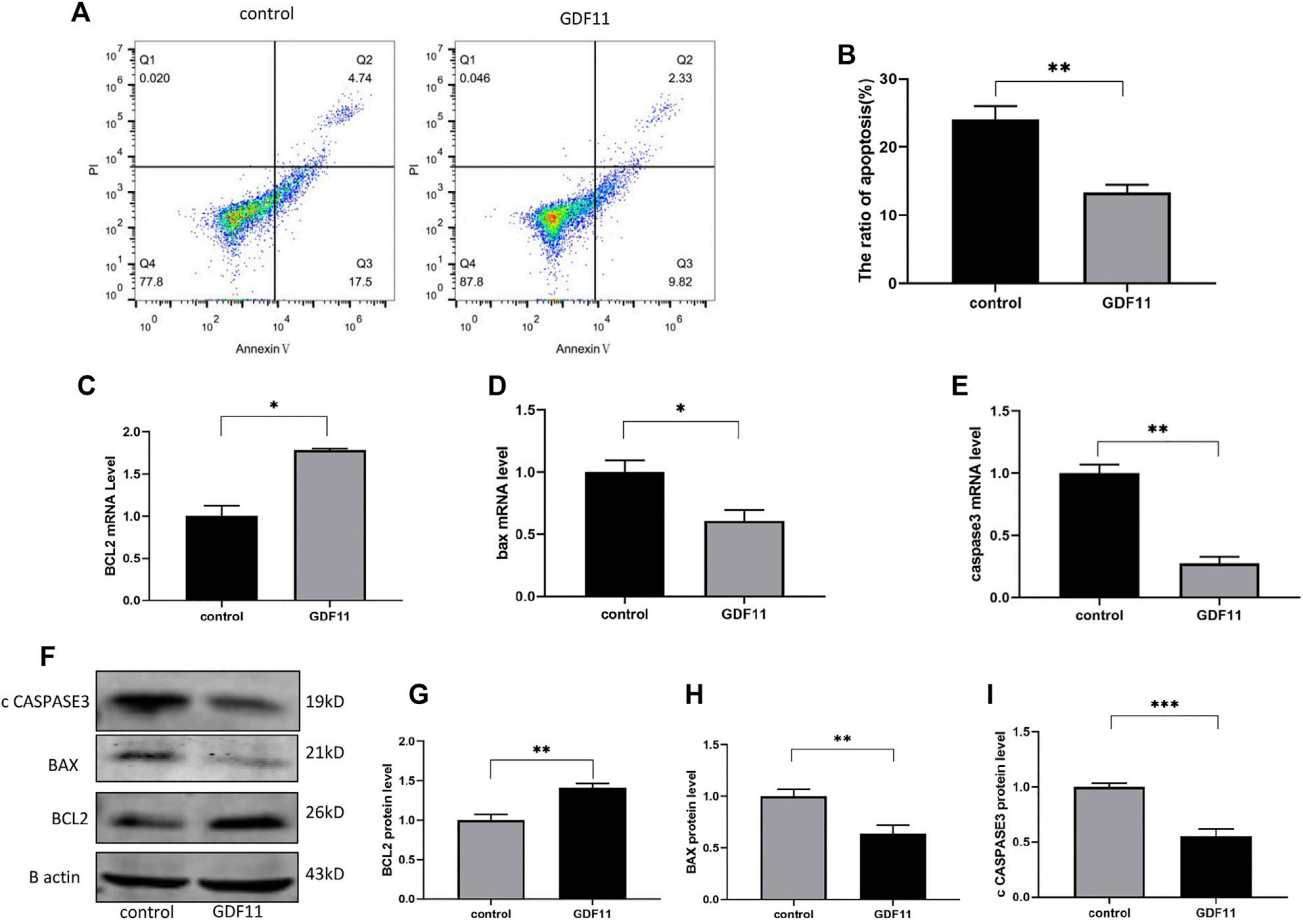
GDF11 inhibited apoptosis of DGR *in vitro*. **(A and B).** Flow cytometry was used to detect the degree of apoptosis of DRG after cultured with recombinant GDF11 for 3 days. **(C, D and E).** DRG were cultured with recombinant GDF11 for 3 days, and mRNA expression of apoptosis-related genes Bcl2, Bax and caspase3 were detected by RT-PCR. **(F, G, H and I).** After DRG were cultured with recombinant GDF11 for 3 days, the protein expressions of apoptosis-related genes Bcl2, Bax and caspase3 were detected by Western blot. Statistical analysis was based on at least three different biological samples and three technical replicates, *p* < 0.05 was considered to be statistically significant.

To investigate the influence of GDF11 on neuronal cells, DRG cells were isolated from rats and cultured with neuronal selective medium to simulate the effect of GDF11 on neuronal cells *in vitro*. One day after the isolation and culture of DRG cells, we added or did not add 10 ng/ ml recombinant GDF11 to the medium, continued to culture the cells for 3 days, and then harvested the cells. Flow cytometry was used for the detection of cell apoptosis. Basic cell apoptosis occurred under normal culture conditions, whereas the apoptotic rate of the cells cultured with GDF11 was significantly decreased (Figures 2A,B). To further confirm the inhibitory effect of GDF11 on cell apoptosis, we detected the expression of genes and proteins in the GDF11 and control groups by Western blotting and qPCR, and the results showed that Bcl2 expression was significantly higher in the GDF11 group than in the control group (Figures 2C,F,G), whereas Bax and caspase3 expression was significantly decreased (Figures 2D–F,H,I); in addition, the results obtained for mRNA and protein expression showed the same trend. These results indicate that GDF11 can regulate the expression of DRG cell apoptosis-related genes and then inhibits DRG cell apoptosis.

### GDF11 Promotes Axonal Growth of DRG Cells *in vitro*

**FIGURE 3:**
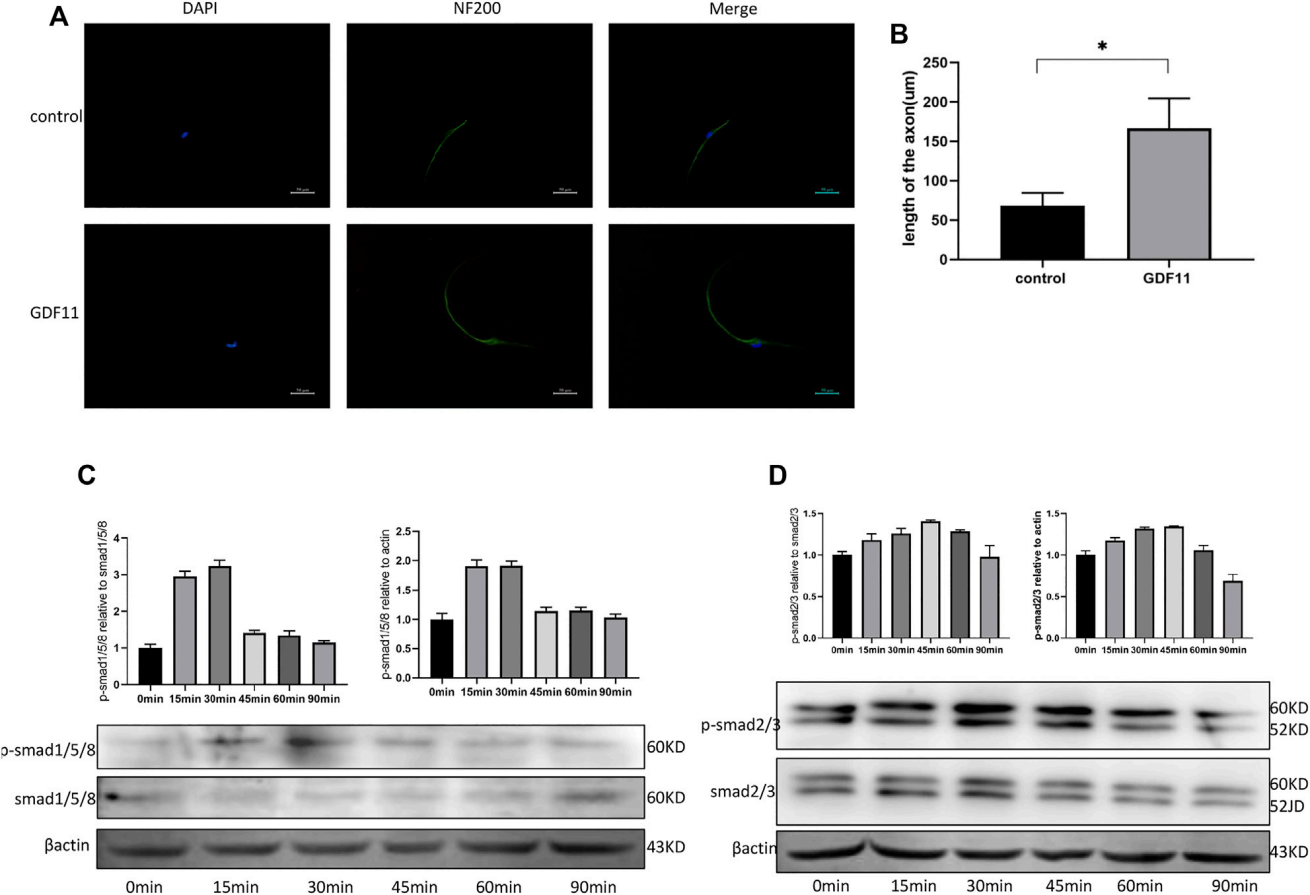
GDF11 promotes axonal growth of DRG cells *in vitro*. **(A and B).** After DRG cells were cultured with recombinant GDF11 for 7 days, axon length was measured by NF200 immunofluorescence. **(C).** Western analysis showed increased phosphorylation of Smad1/5/8 when DRG cells were treated with GDF11. **(D).** Western analysis showed increased phosphorylation of Smad2/3 when DRG cells were treated with GDF11. Statistical analysis was based on at least three different biological samples and three technical replicates, *p* < 0.05 was considered to be statistically significant. Representative images are shown.

To explore the influence of GDF11 on the growth of neuronal axons, DRG cells were isolated and cultured with neuronal selective medium, recombinant GDF11 (10 ng/ ml) protein was added or not added to the medium, and neuronal axons were labeled with NF200. The immunofluorescence results showed that the neuronal axons in the GDF11 group were significantly longer than those in the control group (Figures 3A,B). This result suggests that GDF11 can promote neuronal axon growth. To explore the pathway by which GDF11 acts, phosphorylation of Smad was detected at different time points. The results showed that the phosphorylation of Smad2/3 and Smad1/5/8 was significantly activated after treatment with recombinant GDF11, suggesting that GDF11 may play a role through Smad signaling pathway (Figures 3C,D).

### GDF11 Promotes the Recovery of Damaged Sciatic Nerve Function in Rats

**FIGURE 4:**
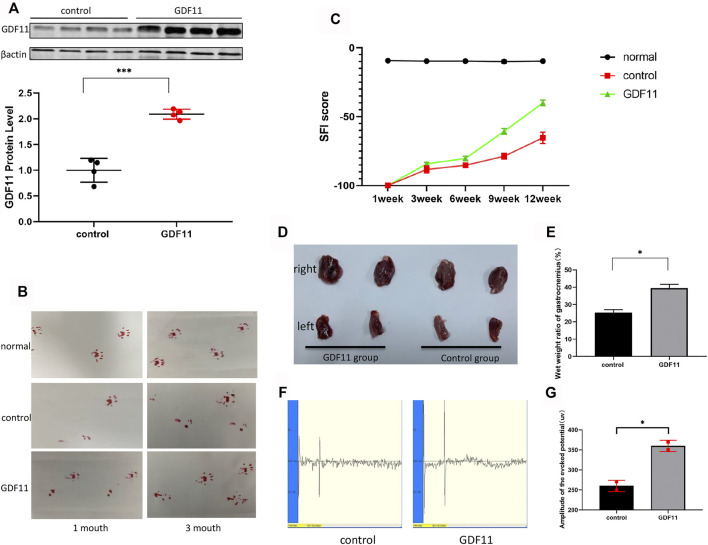
GDF11 promotes the recovery of damaged sciatic nerve function in rats. **(A).** One month after surgery, the sciatic nerves of four rats in each group were randomly selected, and the expression of GDF11 was detected by Western blot. **(B).** The recovery of sciatic nerve function was observed by foot print test at 1 and 3 months after operation. **(C).** SFI value was used to evaluate the recovery of sciatic nerve function in rats at different time points (*n* = 3). **(D and E).** Gastrocnemius atrophy of rats 3 months after operation (*n* = 3). **(F and G).** The nerve conduction function of rat sciatic nerve was measured by action evoked potential (MEP) at 3 months after operation (*n* = 3). Statistical analysis was based on at least three different biological samples and three technical replicates, *p* < 0.05 was considered to be statistically signiandfilig;cant. Representative images are shown.

Based on previous functional studies of GDF11 and our above-described findings, we explored whether continuous delivery of GDF11 *in vivo* could promote restoration of the normal function of the damaged peripheral nervous system, and we constructed a lentiviral vector expressing GDF11. Twenty microliters of control lentivirus and 20 µL of lentivirus-GDF11 were injected at the nerve transection sites in the rats belonging to the control and experimental groups, respectively, immediately after surgery. One month later, four random sciatic nerves from each group were collected for the extraction of proteins from the site of injury in the rats, and the expression of GDF11 was then detected. The results showed that the expression of GDF11 in the lentiviral GDF11 group was significantly higher than that in the control group (Figure 4A). One month after surgery, the affected lateral footprints in the control and GDF11 groups were significantly less likely to land than those in the normal and normal groups (a slight improvement was observed in the GDF11 group). Three months after surgery, we detected the footprints again and found that the abduction and status of the affected lateral footprints in the GDF11 group were better than those in the control group (Figure 4B). For detection of the nerve conduction function of the rats, we assessed the motion-evoked potential of the rats 3 months after the operation, and the results showed that the detection potential of the GDF11 group exhibited a larger amplitude and a shorter latency than that of the control group (Figures 4F,G). We executed the rats immediately after this assessment and carefully stripped the fresh gastrocnemius muscle. The weighing of the gastrocnemius muscle revealed that the ratio of the affected to the healthy side of the gastrocnemius muscle in the GDF11 group was significantly higher than that in the control group (Figures 4D,E), which further confirmed restoration of sciatic nerve impulse conduction and muscle dominance. In addition, we recorded the recovery of the sciatic nerve function in the rats based on the SFI score on a weekly basis for a period of 3 months after surgery. The experimental results showed that the SFI score of the rats with GDF11 overexpression was higher than that of the control group at the same time point, which indicated that the rats belonging to the former group exhibited better recovery of sciatic nerve function (Figure 4C). These experiments suggest that GDF11 overexpression can promote the functional recovery of the transverse sciatic nerve.

### GDF11 Promotes Regeneration of Damaged Sciatic Nerves in Rats

**FIGURE 5:**
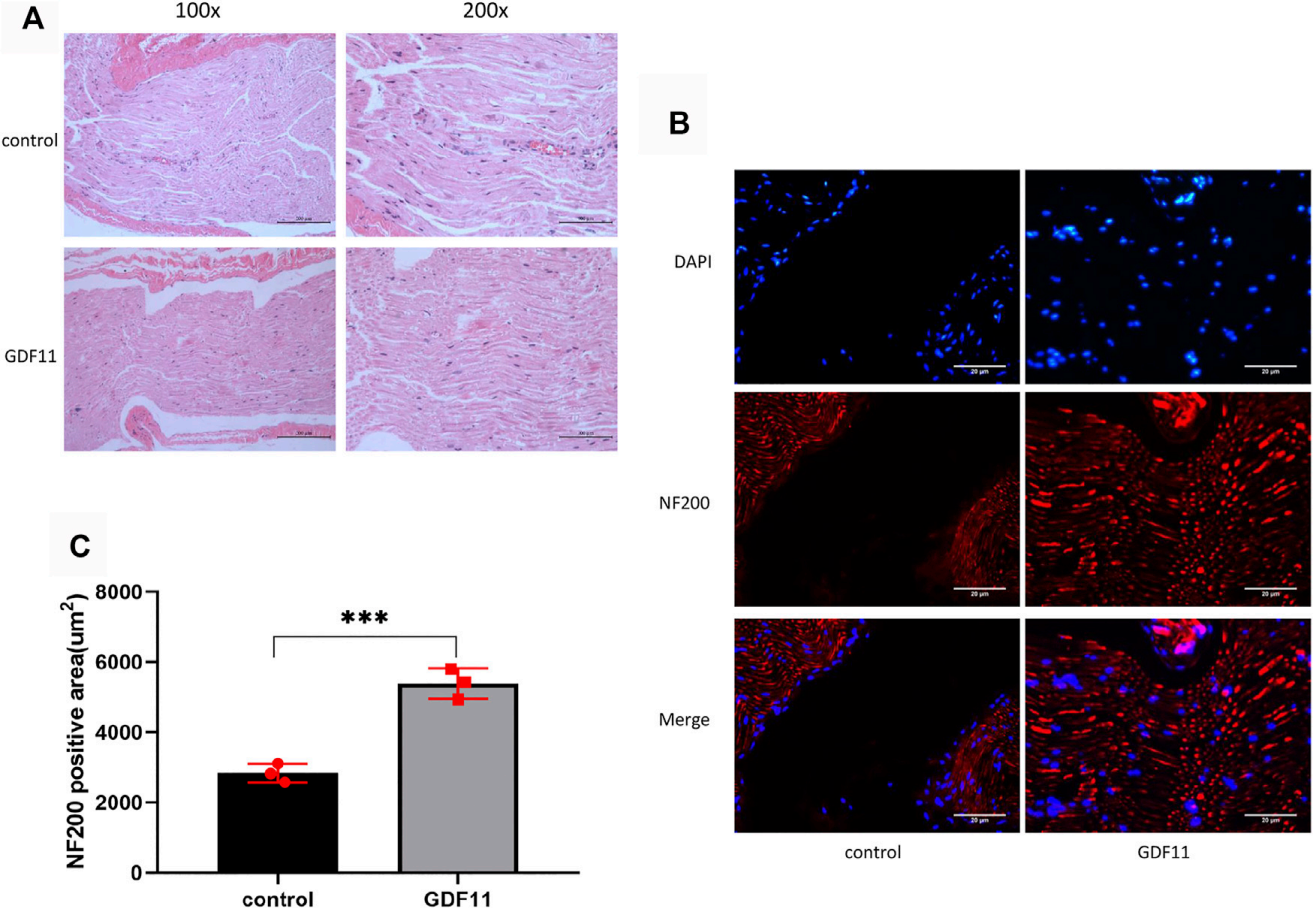
GDF11 promotes regeneration of damaged sciatic nerve in rats. **(A).** HE staining was used to observe the recovery of rat sciatic nerve 3 months after operation (*n* = 3). **(B and C).** The nerve fibers were labeled with NF200 and the nerve growth was detected by immunofluorescence (*n* = 3). Statistical analysis was based on at least three different biological samples and three technical replicates, *p* < 0.05 was considered to be statistically signiandfilig;cant. Representative images are shown.

In the following experiment, we performed HE staining to observe the morphological characteristics of the sciatic nerve. The results showed that the nerve fibers in the control group were disorganized and had little nerve growth, whereas the nerve fibers in the GDF11 group had more nerve growth, were closely arranged and showed a compact structure and regular shape in the lesion area (Figure 5A). We then stained the tissue with NF200, and the results showed that the nerve fibers in the control group were sparse and that almost no nerve growth in the transverse region. The nerve fibers in the GDF11 group were closely arranged, grew at the cross-section (Figures 5B,C), which was consistent with the HE staining results. These findings indicate that GDF11 promotes the growth of nerve fibers.

### GDF11 Promotes Myelin Regeneration of Damaged Sciatic Nerves in Rats

**FIGURE 6:**
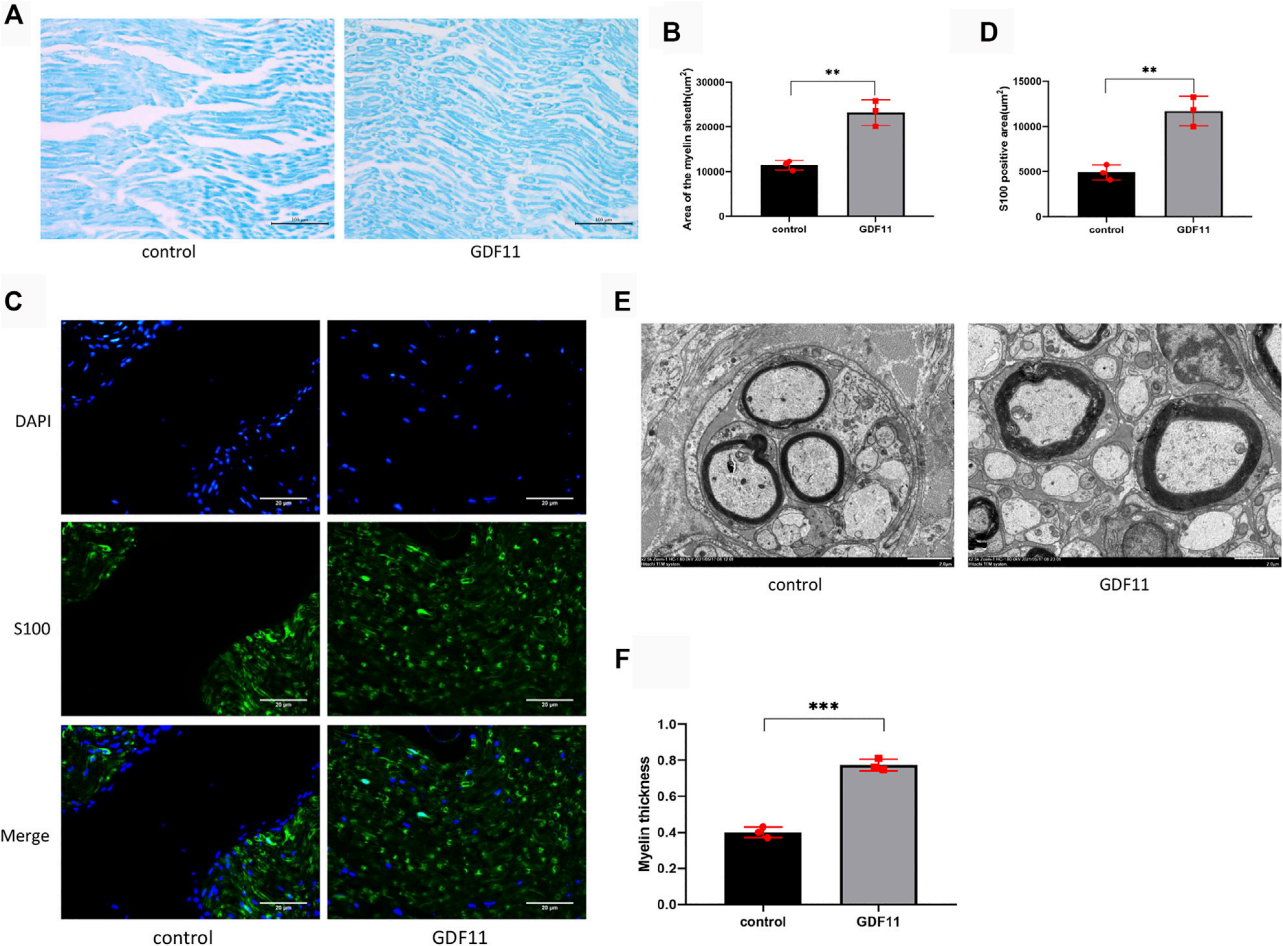
GDF11 promotes myelin regeneration of damaged sciatic nerve in rats. **(A and B).** At 3 months postoperatively, myelin staining was used to assess the extent of myelination in the injured sciatic nerve area in rats (n = 3). **(C and D).** Three months after surgery, S100 was labeled with Schwann cells to detect Schwann cells in the damaged area (n = 3). **(E and F).** Three months after surgery, a transmission electron microscope was performed on the sciatic nerve in the injured area of rats, and myelin sheath thickness and neovascularization were assessed (n = 3). Statistical analysis was based on at least three different biological samples and three technical replicates, *p* < 0.05 was considered to be statistically signiandfilig;cant. Representative images are shown.

Neural function cannot be separated from the normal myelin structure. To clarify the influence of GDF11 on the myelin sheath of nerve fibers, the myelin sheath of the sciatic nerve was subjected to myelin staining, and the results showed the appearance of a large number of areas with myelin staining in the new junction area in the GDF11 group, whereas the control group showed fewer myelin sheath-stained areas in the cross section (Figures 6A,B), which indicated that GDF11 exerted an obvious promoting effect on myelin regeneration. We then labelled Schwann cells with S100 and found that the stained areas of Schwann cells in the GDF11 group were more abundant and formed links (Figures 6C,D), which indicated that GDF11 promotes the proliferation of Schwann cells. In further experiments, we performed electron microscopy to observe the ultramicroscopic structure of the sciatic nerve. The results showed that the myelin sheath in the GDF11 group was more regular and thicker than that in the control group (Figures 6E,F). These studies suggest that GDF11 could promote myelination of the transverse sciatic nerve.

### GDF11 Exerts a Protective Effect on Neuronal Cells in Rats

**FIGURE 7:**
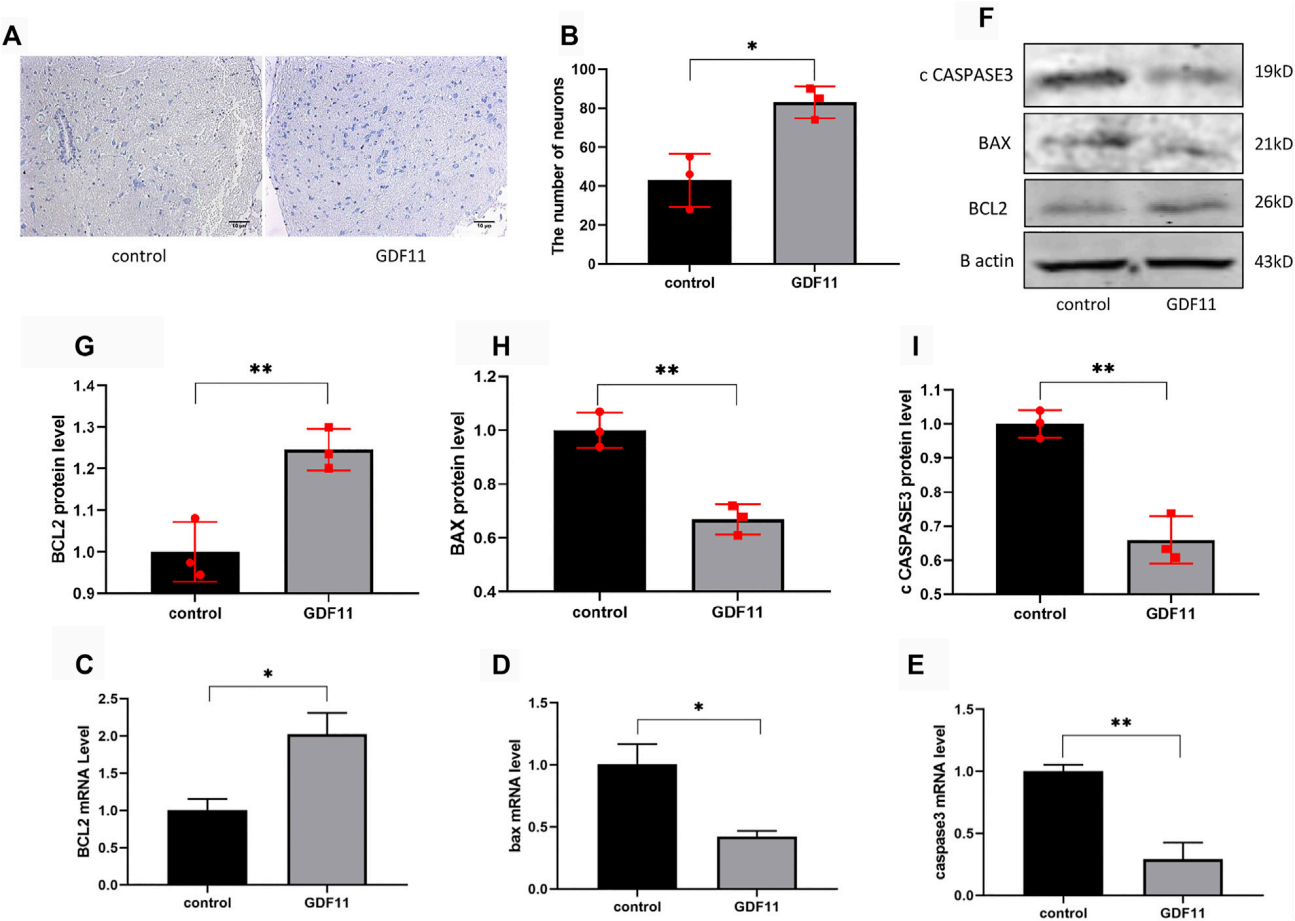
GDF11 has a protective effect on neuronal cells in rats. **(A and B).** Three months after surgery, spinal cords of the rats were sected for Nissl staining to assess the number of neurons alive (*n* = 3). **(C, D and E).** Three months after surgery, the spinal cord of the rats was extracted for RNA, and the mRNA levels of apoptosis-related genes were detected by RT-PCR(*n* = 3). **(F, G, H and I).** Three months after surgery, the spinal cord of the rats was extracted for proteins, and then Western blot was used to measure the protein levels of apoptosis-related genes (*n* = 3). Statistical analysis was based on at least three different biological samples and three technical replicates, *p* < 0.05 was considered to be statistically signiandfilig;cant. Representative images are shown.

The sciatic nerve and spinal cord segments have many contacts. To further investigate whether local injection of lentiviral GDF11 into the sciatic nerve can protect the spinal neurons of corresponding segments, we obtained slices of the spinal cord of rats 3 months after surgery and then detected the neuronal state by Nissl staining. The results showed that the number of neurons in the GDF11 group was higher than that in the control group (Figures 7A,B). These results suggest that transection of the sciatic nerve induces death of the corresponding spinal neurons due to loss of neurotrophic effects, and the overexpression of GDF11 can promote survival of the corresponding neurons. Because *in vitro* cell experiments showed that GDF11 inhibits apoptosis, we explored whether the overexpression of GDF11 could also play the same role *in vivo*. The results showed that the expression of apoptosis-related genes was decreased in the GDF11 group (Figures 7D–F,H,I), but the expression of the apoptosis-related gene Bcl2 was increased (Figures 7C,F,G). The above-mentioned experimental results suggest that GDF11 overexpression *in vivo* could inhibit apoptosis and thus protect sciatic nerve function.

## Discussion

Our study demonstrated that GDF11 can inhibit DRG cell apoptosis and promote axon growth *in vitro*. In addition, the *in vivo* continuous delivery of GDF11 via lentivirus promoted functional recovery of the transected sciatic nerve.

Transection of the peripheral nerve changed the microenvironment of the lesion, and this effect was followed by degeneration of the nerve cells (Sun et al., 2016; Yan et al., 2018; Liu et al., 2019; Liu et al., 2021), deformation and apoptosis of Schwann cells, degradation of the myelin sheath into debris, and inflammatory activation (Caillaud et al., 2019). Modulation of the damaged microenvironment is an effective treatment, and the use of cytokines has been shown to promote axonal regeneration (Jubran and Widenfalk, 2003). However, there is no cure so far, so it is important to explore new therapeutic strategies and their potential mechanisms. Many previous studies suggested promoting the outgrowth of the neuronal axons is important for the maturation and function of the neuron (Guo et al., 2019; Guo R. et al., 2020; Hu et al., 2021; Wei et al., 2021). Our *in vitro* experiments showed that recombinant GDF11 can promote growth of the neuronal axons of DRG cells. Studies have also shown that GDF11 can promote neural stem cell axon growth and even retinal ganglion cell dendrites. Wang et al. showed that GDF11 can induce apoptosis and suppress migration in a dose-dependent manner (Wang Z. et al., 2018), but some studies suggest that GDF11 can inhibit apoptosis (Song et al., 2019; Xiao et al., 2021). In our current study, we treated DRG cells with recombinant GDF11 and found that GDF11 could inhibit DRG cell apoptosis. Previous studies have shown that the TGF family, including GDF11, plays a role through the Smad signaling pathway (Hofer and Schwab, 2019; Luo et al., 2019). Although, the receptor and specific mechanism of GDF11 on neurons should be further explored in future studies, in our study, we demonstrated that GDF11 activates Smad2/3 and Smad1/5/8. Therefore, we hypothesized that GDF11 promotes axon growth and inhibits apoptosis through the Smad signaling pathway, thereby promoting the recovery of damaged sciatic nerve function. Although GDF11 has recently been reported to play an anti-inflammatory role in RA by antagonizing NFκB pathway (Li W. et al., 2019), the role of NFκB in the nervous system remains to be further studied. In addition, some studies have shown that the lentiviral vector overexpression system can continuously express target genes *in vivo* and promote the recovery of neurological function (Hu et al., 2005). The *in vivo* overexpression of GDF11 by viral vectors has been proven to be very helpful for the treatment of many diseases (Dai et al., 2020; Ren et al., 2021). Considering the beneficial effects of lentivirus, a lentiviral vector was used in this study for the *in vivo* overexpression of GDF11 and found that the rats with GDF11 overexpression had higher SFI scores than the control group, which indicated that the sciatic nerve function of the GDF11 group exhibited improved recovery. The detection of action-evoked potentials revealed that the nerve conduction function of the rats in the GDF11 group was better. Action-evoked potentials are an important method for the detection of nerve conduction function, and some previous studies have also indicated that a higher amplitude of action-evoked potentials indicates better nerve conduction function (Zhao et al., 2014). In addition, the wet weight of the gastrocnemius muscle was measured at 3 months, which was consistent with previous studies (Salehi et al., 2019). The degree of atrophy on the diseased side of the gastrocnemius muscle in the GDF11 group, which exhibited better functional recovery, was less severe than that in the control group. This finding also confirms that GDF11 treatment significantly improves recovery of the neurotrophic effects of the sciatic nerve on the muscle that it innervates to a certain extent. However, the environment inside the lesion is complex, thus, the restoration of sciatic nerve function is not simply due to a single cell type (do Carmo Oliveira et al., 2021).

Previous studies have also shown that GDF11 is widely expressed in both neurons and glial cells (Liang et al., 2014), and some studies have shown that GDF11 affects neurogenesis and modulates neurons morphology (Gokoffski et al., 2011; Augustin et al., 2017). After *in vivo* delivery of GDF11 using lentiviral vectors, our results indicate that regardless of the cell in which GDF11 is expressed, the focal area will be a high GDF11 environment, a microenvironment conducive to neural recovery. the HE staining and NF200 labeling of nerve fibers revealed that the overexpression of GDF11 was beneficial to the regeneration of nerve fibers. The recovery of nerve function depends on the myelin sheath structure outside the nerve axon. Thus, we used myelin staining and S100-labeled Schwann cells to confirm that GDF11 overexpression can indeed promote the formation of extra-axonal myelin structures. Previous studies have shown that the growth of neuronal axons promotes myelination, and the existence and migration of Schwann cells guide neuronal axons (Chen et al., 2019). Therefore, we hypothesize that GDF11 promotes the growth of nerve fibers, which in turn leads to Schwann cell proliferation and myelin formation. Further analysis of the submicroscopic structure of most tissues by electron microscopy revealed that GDF11 overexpression resulted in a thicker myelin sheath. Interestingly, we also found that the GDF11 group showed more new blood vessels, whereas previous studies have shown that GDF11 can promote neurovascular regeneration. Neurovascular regeneration can provide a good blood supply for nerve regeneration (Merolli et al., 2009; Goedee et al., 2013). However, whether there is a receptor for GDF11 on Schwann cells, whether GDF11 delivered *in vivo* can directly act on Schwann cells and its potential mechanism will be further explained in the future.

Our results showed that GDF11 inhibits apoptosis both *in vivo* and *in vitro*, and the corresponding number of neurons in the spinal cord segment was higher, which might have been observed because stimulation of the nerve in the injured sciatic nerve focal area leads to apoptosis of the corresponding neurons. In fact, the injured sciatic nerve terminal is very sensitive to the injured environment and synthesizes some special signaling molecules to reverse transport to the neuronal cell body. When the neuronal cell body receives signal stimulation, it initiates the transcription of apoptotic genes (Yudin et al., 2008), and delivery of GDF11 improves the microenvironment at the site of damage. GDF11 can protect neurons from apoptosis Correspondingly, good spinal cord function is more positively important for the nutrition and innervation of nerve fibers, and a variety of complex factors promote improved recovery of sciatic nerve function.

In general, based on the present study, we conclude that GDF11 promotes axonal growth and inhibits DRG cell apoptosis *in vitro*. In addition, the *in vivo* overexpression of GDF11 by lentivirus can promote the recovery of sciatic nerves after transection by promoting axonal growth and inhibiting neuronal apoptosis in the spinal cord.

## Data Availability

The original contributions presented in the study are included in the article/supplementary material, further inquiries can be directed to the corresponding authors.
